# Immunohistochemical Analysis of Histone H3 Modification in Newt Tail Tissue Cells following Amputation

**DOI:** 10.1155/2021/8828931

**Published:** 2021-01-05

**Authors:** Ji-Wen Wu, Xu Zhang, Reiko Sekiya, Kiyoshi Aoyagi, Tao-Sheng Li

**Affiliations:** ^1^Department of Stem Cell Biology, Atomic Bomb Disease Institute, Nagasaki University, Nagasaki 852-8523, Japan; ^2^Department of Human Anatomy, Histology and Embryology, School of Basic Medical Sciences, Fujian Medical University, Fuzhou 350108, China; ^3^Department of Public Health, Nagasaki University Graduate School of Biomedical Sciences, Nagasaki 852-8523, Japan

## Abstract

**Background:**

Newts have impressive regenerative capabilities, but it remains unclear about the role of epigenetic regulation in regeneration process. We herein investigated histone modifications in newt tail tissue cells following amputation.

**Methods and Results:**

Iberian ribbed male newts (6-8 months old) were suffered to about 1.5 cm length of amputation of their tails for initiating regeneration process, and the residual stump of tail tissues was collected for immunohistochemical analysis 3 days later. Compared to the tissue cells of intact tails, c-kit-positive stem cells and PCNA-positive proliferating cells were significantly higher in tails suffered to amputation (*P* < 0.001). Amputation also significantly induced the acetylation of H3K9, H3K14, and H3K27 in cells of the tails with amputation (*P* < 0.001), but did not significantly change the methylation of H3K27 (*P* = 0.063).

**Conclusion:**

These results suggest that epigenetic regulation likely involves in newt tail regeneration following amputation.

## 1. Introduction

Tissue regeneration capabilities are of great importance in many organisms; however, different organisms have different tissue regeneration abilities [1]. Differed from mammalians, amphibians remain significant regenerative abilities [2]. For example, newts can completely regenerate the tail and limbs after amputation.

Regeneration is a very complex process, including the proliferation, migration, dedifferentiation, and transdifferentiation of tissue cells, as well as the proliferation, differentiation, and maturation of resident tissue stem/progenitor cells. It is still poorly understood the molecular mechanisms underlying the regeneration process in newts [3, 4], especially the role of epigenetic modifications of histone and chromatin.

Epigenetic modifications include acetylation, methylation, phosphorylation, ubiquitylation, and sumoylation. Histone acetylation, one of the most popular epigenetic events, has been demonstrated to involve in diverse biological processes, including the regeneration of injured tissues [5]. Histone H3 is one of the most extensively modified histone out of the five primary histone proteins. In most species, histone H3 is primarily acetylated at lysines 9 (H3K9), 14 (H3K14), and 27 (H3K27). It has been reported that the acetylated H3K9, H3K14, and H3K27 are enriched near the transcription start sites [6]. Acetylated H3K9 has been observed in actively transcribed promoters, which associated with active transcription [7]. Acetylated H3K27 has also been defined as an active enhancer during nuclear reprogramming [8]. However, it has been reported the decreased expression of acetylated H3K9 in newt during lens regeneration [4]. Therefore, it keeps unclear on the reality and potential role of histone modifications during regenerative process in newt.

Using a tail amputation model for initiating the regenerative process, we herein investigated the histone modifications in tissue cells of adult newts. We confirmed a significant increase of acetylated H3K9, H3K14, and H3K27 in tail tissue cells at 3 days after amputation.

## 2. Materials and Methods

### 2.1. Newts, Tail Amputation, and Tissue Preparation

Iberian ribbed newts were obtained from the Tottori University [9]. Nine male adult newts (6-8 months old, 28.4 ± 1.7 g) were used for the experiment. This study was approved by the Institutional Animal Care and Use Committee of Atomic Bomb Disease Research Institute, Nagasaki University (#2017-1). All animal procedures were performed in accordance with the institutional and national guidelines.

Newts were anesthetized by soaking in 0.2% MS-222 (Tokyo Chemical Industry, Japan) for 15 min, followed by amputating about 1.5 cm length of the tails (Supplemental Figure 1). All male newts bleed in a room with serve temperature control (25 ± 1.5°C). Soon after amputation, we returned these newts back to their daily living water bath.

The regenerating tissues were harvested from the residual stump of distal part of tails 3 days after amputation (amputated group, *n* = 9). As an intact control, we also collected the resected tail tissues of proximal part (intact group, *n* = 9). The tissues were immediately fixed in 4% paraformaldehyde for 24 h and embedded in paraffin (Supplemental Figure 1). Sections of 5 *μ*m thick were used for immunohistochemical analysis.

### 2.2. Immunohistochemical Analysis

Paraffin sections were deparaffinized and then incubated with blocking solution (1% BSA in PBS) at room temperature for 30 min. After blocking, the sections were incubated with rabbit anti-Histone H3 (acetyl K9) monoclonal antibody (Abcam; 1 : 1000 dilution), rabbit anti-Histone H3 (acetyl K14) monoclonal antibody (Abcam; 1 : 500 dilution), rabbit anti-Histone H3 (acetyl K27) monoclonal antibody (Abcam; 1 : 1000 dilution), mouse anti-Histone H3 (di methyl K27, tri methyl K27) monoclonal antibody (Abcam; 1 : 500 dilution), rat anti-mouse PCNA monoclonal antibody (Santa Cruz Biotechnology; 1 : 100 dilution), and rat anti-mouse CD117/c-kit monoclonal antibody (R&D Systems; 1 : 100 dilution), respectively, for overnight at 4°C. The sections were washed three times with PBS and then incubated with the appropriate secondary antibodies conjugated with Alexa Fluor® 488, Alexa Fluor® 546, or Alexa Fluor® 594 for 60 min at room temperature. The specificity of all primary antibodies was confirmed by either positive control (mouse bone marrow and liver tissues) or negative control (with second antibodies alone). After washing three times with PBS, the nuclei were labelled with DAPI (Invitrogen).

Positively stained cells were detected under the confocal laser scanning microscopy (FV10i-LIV, Olympus), and digital images were acquired using FV10-ASW software (Olympus) with a 60-fold magnification lens. Two sections from each tissue sample were stained for every primary antibodies. Ten images were randomly acquired from the two stained slides of each tissue sample for quantitative analysis. The PCNA- and c-kit-positive cells were counted by the same threshold (Max. 255, Min.106). However, histone modifications were evaluated by measuring the signal intensities of immunostainings and normalized by DAPI using Image J (ver1.8.0, NIH). To reduce the technical variation, we tried our best to standardize each experimental step.

### 2.3. Statistical Analysis

All the results are presented as the mean ± SD. Unpaired two-tailed *t*-test was used for statistical analyses. All analyses were carried out with the SPSS19.0 statistical software (IBM SPSS Co., USA). *P* < 0.05 was considered statistically significant.

## 3. Results

### 3.1. Increased Number of PCNA-Positive Proliferating Cells and c-Kit-Positive Stem Cells around the Residual Stump of Tail after Amputation

**Figure 1:**
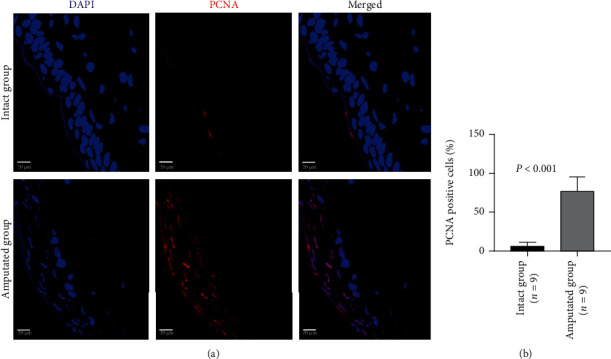
Immunohistochemical analysis on the PCNA-positive proliferating cells in tails 3 days after amputation. Representative images (a) and quantitative data (b) show the PCNA-positive cells in intact tails and amputated tails. Scale bar: 20 *μ*m.

**Figure 2:**
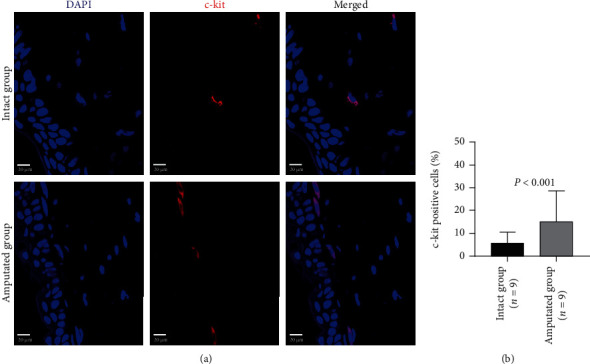
Immunohistochemical analysis on the c-kit-positive stem cells in tails 3 days after amputation. Representative images (a) and quantitative data (b) show the c-kit-positive stem cells in intact tails and amputated tails. Scale bar: 20 *μ*m.

To confirm the initiation of regeneration, we investigated the PCNA-positive proliferating cells and c-kit-positive stem cells around the residual stump of tail tissues 3 days after amputation by immunohistochemistry staining (Supplemental Figure 1). The majority (up to 70%) of cells in tail tissues were positively stained by PCNA at 3 days after amputation (Figure 1). Compared with the intact tail tissues, the percentage of PCNA-positive cells were dramatically increased around the residual stump of tail tissues 3 days after amputation (5.63 ± 5.40 vs. 76.80 ± 18.79, *P* < 0.001, Figure 1). Moreover, the c-kit-positive stem cells were rarely detected in the intact tail tissues, but more frequently detected around the residual stump of tail tissues 3 days after amputation (Figure 2). Quantitative data showed that the percentage of c-kit-positive stem cells was significantly higher in the amputated group than that of the intact group (5.57 ± 4.97 vs. 14.87 ± 13.81, *P* < 0.001, Figure 2). In contrast to the wide distribution of PCNA-positive cells in epidermis, these c-kit-positive cells were almost found in the stratum basale epidermis.

### 3.2. Amputation Significantly Induced the Acetylation of H3K9, H3K14, and H3K27, but Did Not Significantly Change the Methylation of H3K27

**Figure 3:**
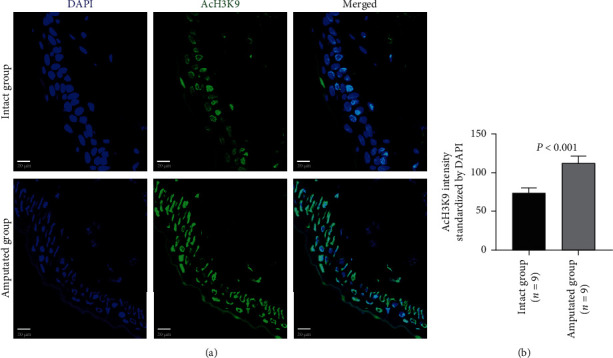
Immunohistochemical analysis on the acetylation of H3K9 in tissue cells 3 days after amputation. Representative images (a) and quantitative data (b) show the expression of acetylated H3K9 (AcH3K9) in intact tails and amputated tails. Quantitative data was normalized by DAPI. Scale bar: 20 *μ*m.

**Figure 4:**
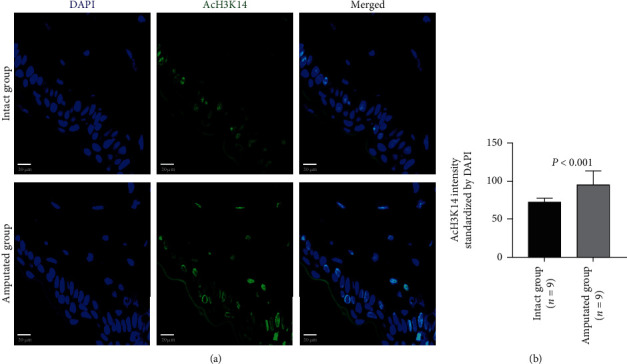
Immunohistochemical analysis on the acetylation of H3K14 in tissue cells 3 days after amputation. Representative images (a) and quantitative data (b) show the expression of acetylated H3K14 (AcH3K14) in intact tails and amputated tails. Quantitative data was normalized by DAPI. Scale bar: 20 *μ*m.

**Figure 5:**
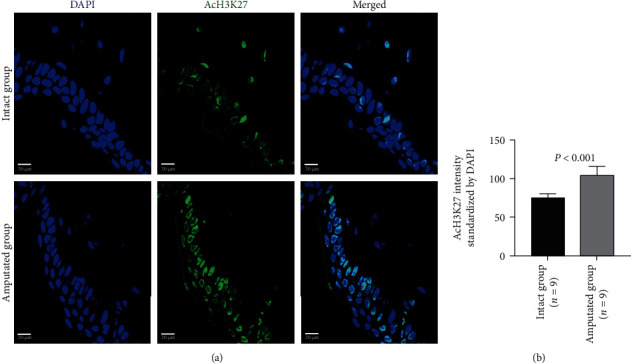
Immunohistochemical analysis on the acetylation of H3K27 in tissue cells 3 days after amputation. Representative images (a) and quantitative data (b) show the expression of acetylated H3K27 (AcH3K27) in intact tails and amputated tails. Quantitative data was normalized by DAPI. Scale bar: 20 *μ*m.

Histone modifications in tail tissue cells were also evaluated by immunohistochemistry staining. The acetylated H3K9, H3K14, and H3K27 could be detected in the nuclear of cells in either intact tails or amputated tails (Figures 3–5, Supplemental Figure 2–4). Compared with the intact group, extensive positive staining was detected in the amputated group. Quantitative data showed that the signal intensity of staining on the acetylated H3K9 (41.00 ± 18.59 vs. 91.40 ± 7.60, *P* < 0.001), H3K14 (44.97 ± 12.77 vs. 70.67 ± 24.70, *P* < 0.001), and H3K27 (54.87 ± 15.11 vs. 73.70 ± 25.37, *P* < 0.001) was significantly higher in the amputated group than the intact group (Figures 3–5; Supplemental Figure 2–4).

**Figure 6:**
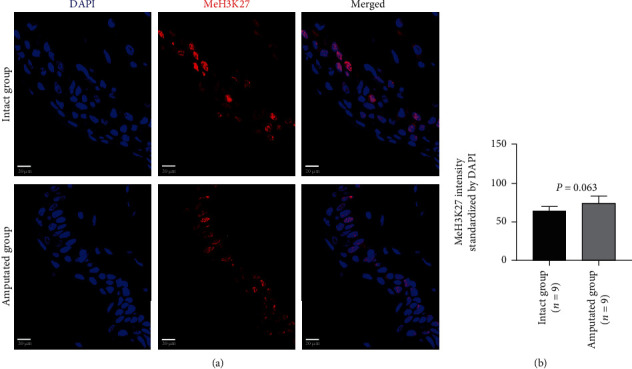
Immunohistochemical analysis on the methylation of H3K27 in tissue cells 3 days after amputation. Representative images (a) and quantitative data (b) show the expression with methylated H3K27 (MeH3K27) in intact tails and amputated tails. Quantitative data was normalized by DAPI. Scale bar: 20 *μ*m.

In contrast to the enhanced expression of acetylated H3K27 around the residual stump of tail tissues 3 days after amputation, the methylation of H3K27 was indicated no significant difference in tissue cells between the intact group and the amputated group (39.63 ± 15.45 vs. 52.40 ± 26.33, *P* = 0.063, Figure 6; Supplemental Figure 5).

## 4. Discussion

Differs from the limited regenerative potency in vertebrates, amphibians, especially the newt, display a more remarkable regenerative potency. However, it has not yet been fully understood the underlying mechanisms on the remarkable regeneration of injured tissues/organs in newt. As epigenetic modifications are known to regulate the proliferation and dedifferentiation of tissue cells, we speculate that histone modifications may also involve in the regenerative process in newts. Using a model of tail amputation, we observed a dramatically increasing of PCNA-positive proliferating cells around the residual stump of tail 3 days after amputation, confirming the robust regeneration of amputated tails. The c-kit-positive stem cells were also significantly increased around the residual stump of amputated tails, suggesting a probable event of dedifferentiation. As expected, immunohistochemical analysis showed that the amputation induced the acetylation of H3K9, H3K14, and H3K27, but did not significantly change the methylation of H3K27.

Regeneration of injured tissues/organs is complex processes [10, 11], including the proliferation and dedifferentiation of remaining tissue cells and the differentiation and maturation of resident stem/progenitor cells. The dramatically increase of PCNA-positive cells indicated the proliferative activity might mainly contribute the regeneration of amputated tails in newts. Dedifferentiation, a biological conversion of differentiated/maturated cells into undifferentiated/immaturated cells, is also known to contribute the regeneration of injured tissues/organs in various species of animals [12, 13]. Although mammalian cells also have the ability to dedifferentiate if given the appropriate triggers [14], dedifferentiation is very commonly found in amphibians. Therefore, dedifferentiation might be induced in the maturated tissue cells to contribute mainly the regeneration of amputated tails.

Previous studies have reported the induction of pluripotent factors during regeneration in newts [15], but it is still kept controversy on the number, origination, and potential role of stem cells in newts [16]. Using a common stem cell marker of c-kit, we could find a few of c-kit-positive cells around the stratum basale epidermis of the intact tail skin tissues suggests there has resident stem cells in newts. However, doubled number of c-kit-positive stem cells were detected around the residual stump of amputated tails comparing to intact tail tissues, and these c-kit-positive stem cells were also mostly detected in the stratum basale epidermis. Therefore, we thought that the increased number of c-kit-positive stem cells could be resulted from the proliferation of resident stem cells. Although there is increased number of stem cells, extensive dedifferentiation of the maturated tissue cells was not observed in amputated tails in this study.

An epigenetic trait is a stably heritable phenotype resulting from changes in a chromosome without alteration in the DNA sequence [17]. Histone acetylation, one of the best characterized epigenetic modifications, is known to increase the expression of gene through transcriptional activation [18]. The epigenetic modifications of histone H3 are known to regulate cellular dedifferentiation and individual development [4], and the acetylation is a good candidate marker to distinguish between active and poised enhancer states [8]. Histone acetylation is generally known to activate the transcription of genes, and the acetylations of H3K9 and H3K27 have been previously demonstrated to correlate with transcriptional activation of chromatin [19, 20]. In this study, we also found more extensive expression of the acetylated H3K9, H3K14, and H3K27 around the residual stump of amputated tails, indicating the potential role of epigenetic modifications in regulating the regeneration process.

Histone methylation is the modification of certain amino acids in a histone protein by the addition of one, two, or three methyl groups. The methylation of H3K27 is usually linked to transcriptional repression [21, 22]. It has been reported that intact zebrafish silenced developmental regulatory genes, but the silenced genes are converted to an active state by losing MeH3K27 modification during fin generation [4]. In contrast to the enhanced expression of acetylated H3K27, our data showed that the methylation of H3K27 was not significantly induced around the residual stump of amputated tails within 3 days. Therefore, we speculated that the methylation of H3K27 unlikely played critical role in initiating the regeneration process in newts soon after injury.

Disagreed with our data, a previous study has reported the increased expression of methylated H3K27 and the decreased expression of acetylated H3K9 for initiating the transdifferentiation of iris cells after lentectomy in newt [23]. It has also reported that DNA methylation ensures gene expression programs for controlling Muller glia cell reprogramming into neurons in zebrafish during retina regeneration [24]. The reason on these different findings among studies keeps unclear because of the high flexibility of epigenetic regulation depending on the systemic and local conditions. Actually, it has reported about dual roles of acetylated H3K9 in stem cell differentiation [25].

There has several limitations on this study. The first, as this study was designed for proof-of-concept, we only performed all investigations at 3 days after amputation. Although it will be largely varied among the species of animals, histone acetylation of resident cells can be quickly induced to initiate the regenerative process after injury. Therefore, the histone acetylation may be induced in the newt tail tissue cells very soon after amputation. Indeed, histone acetylation has been previously reported to be increased within 24 hours in Xenopus tadpole tail after amputation [26]. As we analysed the histone acetylation at only 3 days after amputation, further studies are highly required to uncover the dynamic changes of histone acetylation in the tail tissue cells following amputation in the future. The second, we used immunohistochemistry analysis in this study. That is because we wondered whether histone modification predominantly observed around the marginal regions after amputation. However, other methods, such as Western blot will be better for the quantification of histone modification. Otherwise, it was difficult for us to exactly define the special cell types in the tail tissues for analysis. Although most of the cells positively stained with PCNA, AcH3K9, AcH3K14, and AcH3K27 were uniformly detected at the epidermis of the skin tissue, we failed to confirm the relationship between proliferating cells and histone acetylation by double staining.

In summary, preliminary data from our *in vivo* experiment indicated an enhanced expression of acetylated H3K9, H3K14, and H3K27 in tissue cells around the residual stump of amputated tails. Although in the absence of direct evidence, epigenetic modifications likely involve in regenerating the amputated tails of newts.

## Data Availability

All the data supporting the findings of this study are available within the paper and also are available from the corresponding author upon reasonable request.
