# Supplementary material for: Immunohistochemical Analysis of Histone H3 Modification in Newt Tail Tissue Cells following Amputation
Source: Stem Cells Int. 2021 Jan 5;2021:8828931. doi: 10.1155/2021/8828931 (PMC7806392; doi:10.1155/2021/8828931)

Immunohistochemical analysis of histone H3 modification in newt tail tissue cells following amputation

Jiwen Wu[1,2,3], Xu Zhang[1], Sekiya Reiko[1], Kiyoshi Aoyagi[3], Tao-Sheng Li[1*]

[1] Department of Stem Cell Biology, Atomic Bomb Disease Institute, Nagasaki University, Nagasaki, 852-8523, Japan

[2] Department of Human Anatomy, Histology and Embryology, School of Basic Medical Sciences, Fujian Medical University, Fuzhou, 350108, China

[3] Department of Public Health, Nagasaki University Graduate School of Biomedical Sciences, Nagasaki, 852-8523, Japan

*<u>**Corresponding author:**</u>

Tao-Sheng Li, MD, Ph.D.
Department of Stem Cell Biology,
Atomic Bomb Disease Institute,
Nagasaki University,
1-12-4 Sakamoto, Nagasaki 852-8523, Japan.
Tel: +81-95-819-7099; Fax: +81-95-819-7100. E-mail: litaoshe@nagasaki-u.ac.jp

Supplemental information

Supplementary Figure 1**.** Schematic diagram on tissue sampling for experiments.

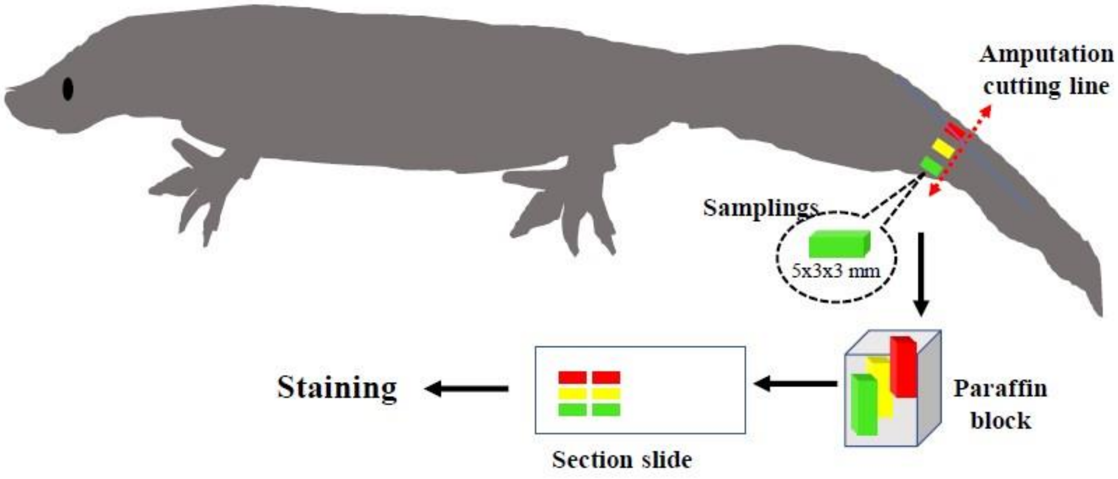

Supplementary Figure 2**.** Immunohistochemical analysis on the expression of acetylated H3K9 (AcH3K9) in tissue cells 3 days after amputation. Representative images display in grayscale.

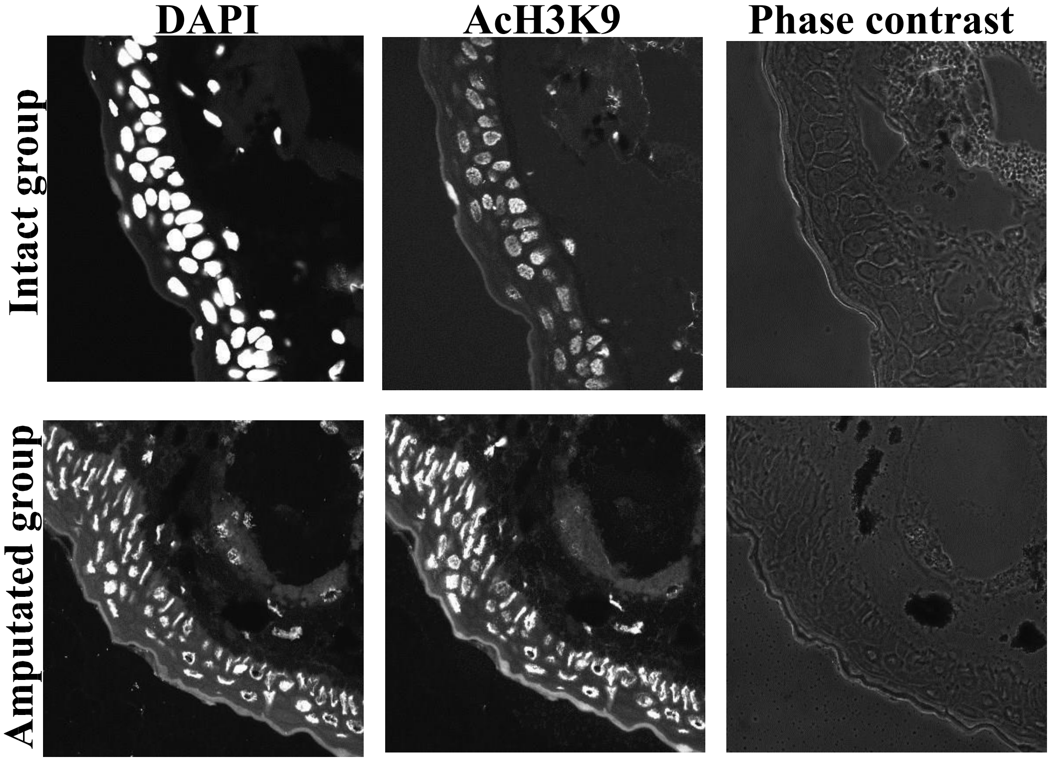

Supplementary Figure 3**.** Immunohistochemical analysis the expression of acetylated H3K14 (AcH3K14) in tissue cells 3 days after amputation. Representative images display in grayscale.

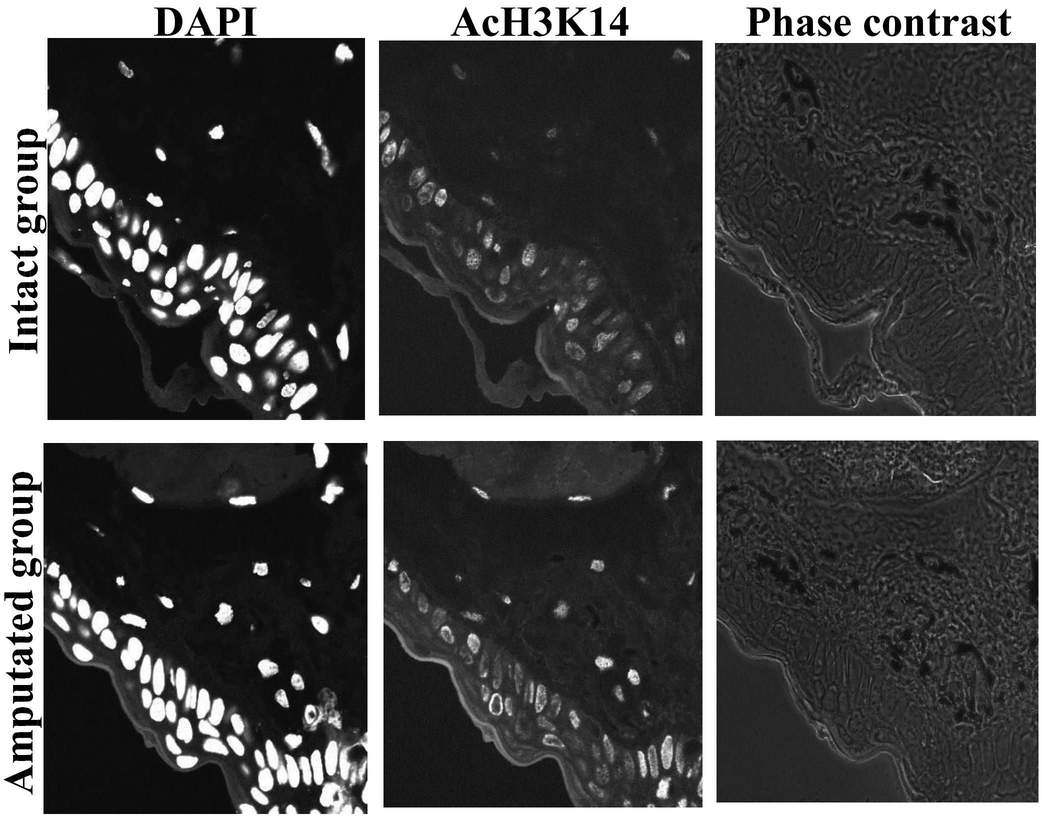

Supplementary Figure 4. Immunohistochemical analysis on the expression of acetylated H3K27 (AcH3K27) in tissue cells 3 days after amputation. Representative images display in grayscale.

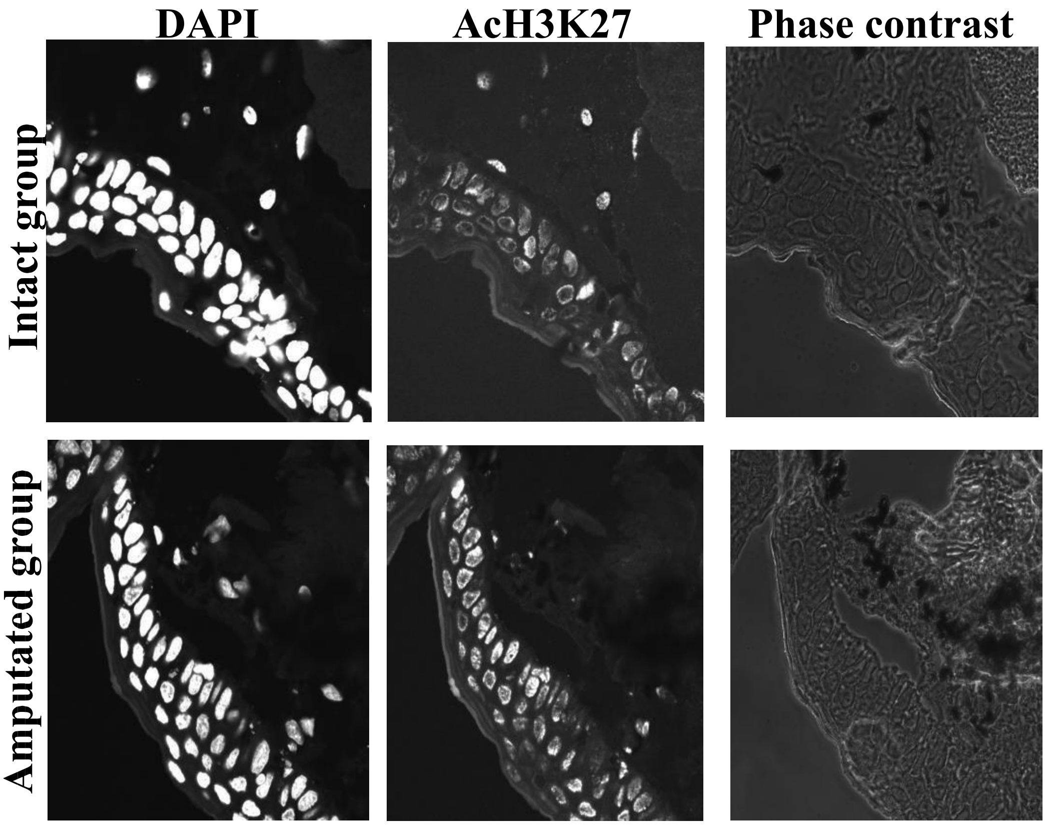

Supplementary Figure 5. Immunohistochemical analysis on the expression of methylated H3K14 (MeH3K27) in tissue cells 3 days after amputation. Representative images display in grayscale.

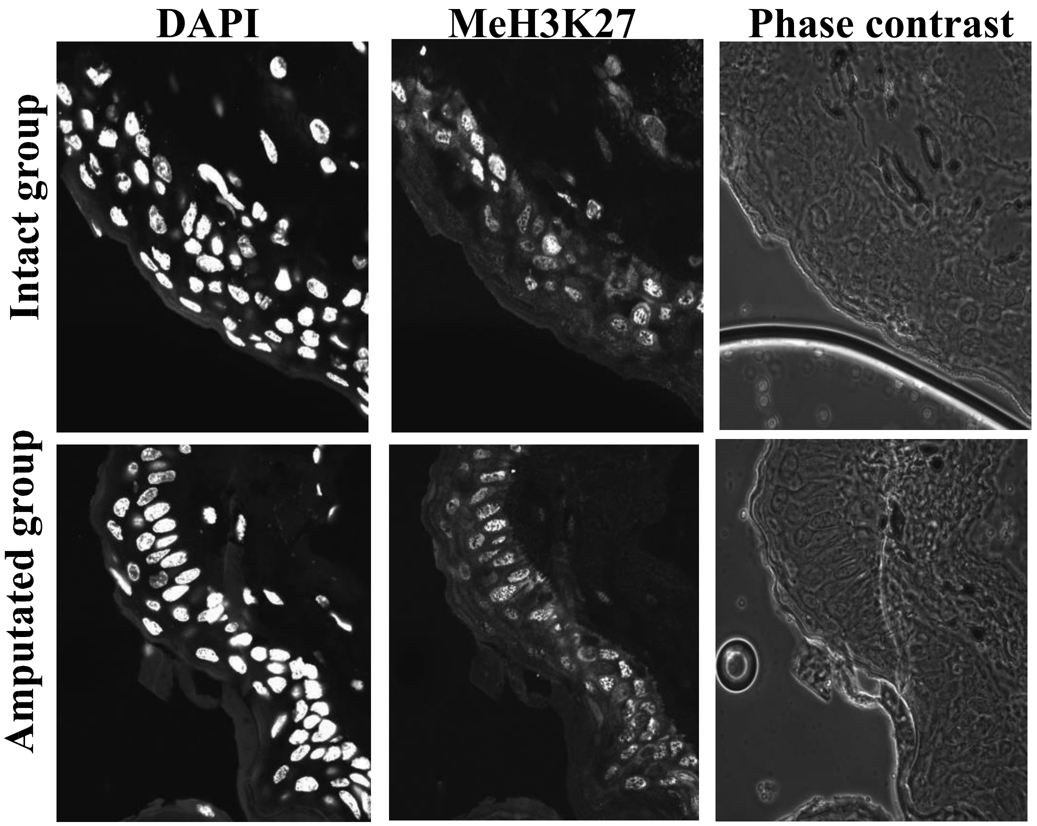

Supplement: Supplementary Materials — Supplementary Figures 1–5. [file 8828931.f1.pdf]
